# Intensity modulated radiotherapy with fixed collimator jaws for locoregional left-sided breast cancer irradiation

**DOI:** 10.18632/oncotarget.16634

**Published:** 2017-03-28

**Authors:** Juanqi Wang, Zhaozhi Yang, Weigang Hu, Zhi Chen, Xiaoli Yu, Xiaomao Guo

**Affiliations:** ^1^ Department of Radiation Oncology, Fudan University Shanghai Cancer Center, Department of Oncology, Shanghai Medical College, Fudan University, 200032, Shanghai, China

**Keywords:** breast cancer, radiotherapy, regional lymph node, IMRT, fixed collimator jaws technique

## Abstract

The purpose of this study is to evaluate the intensity modulated radiotherapy (IMRT) with the fixed collimator jaws technique (FJT) for the left breast and regional lymph node. The targeted breast tissue and the lymph nodes, and the normal tissues were contoured for 16 left-sided breast cancer patients previously treated with radiotherapy after lumpectomy. For each patient, treatment plans using different planning techniques, i.e., volumetric modulated arc therapy (VMAT), tangential IMRT (tangential-IMRT), and IMRT with FJT (FJT-IMRT) were developed for dosimetric comparisons. A dose of 50Gy was prescribed to the planning target volume. The dose-volume histograms were generated, and the paired *t-test* was used to analyze the dose differences. FJT-IMRT had similar mean heart volume receiving 30Gy (V30 Gy) with tangential-IMRT (1.5% and 1.6%, *p* = 0.41), but inferior to the VMAT (0.8%, *p* < 0.001). In the average heart mean dose comparison, FJT-IMRT had the lowest value, and it was 0.6Gy lower than that for the VMAT plans (*p* < 0.01). A significant dose increase in the contralateral breast and lung was observed in VMAT plans. Compared with tangential-IMRT and VMAT plans, FJT-IMRT reduced the mean dose of thyroid, humeral head and cervical esophageal by 47.6% (*p* < 0.01) and 45.7% (*p* < 0.01), 74.3% (*p* = < 0.01) and 73% (*p* = < 0.01), and 26.7% (*p* = < 0.01) and 29.2% (*p* = < 0.01). In conclusion, compared with tangential-IMRT and VMAT, FJT-IMRT plan has the lowest thyroid, humeral head and cervical esophageal mean dose and it can be a reasonable treatment option for a certain subgroup of patients, such as young left-breast cancer patients and/or patients with previous thyroid disease.

## INTRODUCTION

Radiotherapy after breast conservative surgery (BCS) leads to the reduction of any first recurrence and breast cancer mortality [1]. Recent reports of two prospective studies aiming to compare breast radiation alone with breast plus regional lymph node (RLN) radiation which includes internal mammary node (IMN), periclavicular node (supra- and infra-clavicular node) indicate that additional nodal irradiation statistically significantly improves locoregional control and disease free survival [2, 3]. Conventional three-dimensional conformal radiotherapy (3DCRT) was the standard treatment technique [4]. However, the design of treatment plan could be a complicated task especially for comprehensive locoregional radiotherapy as the balance between normal tissue protection and planning target volume (PTV) coverage and dose homogeneity must be achieved [5].

Many sophisticated radiotherapy techniques had been used in the dosimetric study (6–10). Fixed gantry intensity modulated radiotherapy (IMRT), tomotherapy and volumetric modulated arc therapy (VMAT) have shown superior PTV coverage and dose sparing of the heart and the ipsilateral lung. However, in some cases these techniques may place beams pass through the contralateral breast and the contralateral lung which may cause higher doses to those critical structures. With the consideration of the modestly increased risks of radiation induced secondary cancer due to the contralateral breast dose in women under the age of forty years old [11, 12], the tangential multibeam fixed gantry IMRT (tangential-IMRT) technique was used to provide a compromise between PTV coverage and normal tissue sparing [9, 13]. However, the treatment plans in these publications were not designed to evaluate the dose to the organs at risk (OARs) in the periclavicular region such as thyroid, shoulder, and cervical esophagus.

The thyroid which plays a vital role in the body's hormonal milieu is known to be radiosensitive. Hypothyroidism, a common late adverse effect after the irradiation of head and neck cancer or lymphoma [14–19], which has been linked with increased morbidity and mortality [20, 21]. Recently, several authors reported that the thyroid could be exposed to a considerable dose when 3DCRT plans were used in the locoregional breast radiotherapy, and up to 44% of patients were at risk of post radiation thyroid function abnormality [22, 23]. In addition, shoulder symptoms including pain, stiffness and difficulty in raising or moving arm were usually presented in patients treated with axillary lymph node dissection and lymphatic irradiation [24, 25]. From this view, it is necessary to consider avoiding irradiation of thyroid and shoulder in designing IMRT plans for breast treatment, especially for patients with previous thyroid disease.

In conventional IMRT beam intensity is modulated by the multileaf collimator during which the collimator jaw position is automatically set to cover the entire PTV. Recently, a fixed collimator jaws technique (FJT) IMRT was developed where the collimator jaws were locked to a fixed position during optimization for large fields in order to circumvent the problems associated with split fields and to further spare the critical OARs [26, 27]. The collimator jaw positions in FJT IMRT could be adjusted depending on the PTV and OARs positions, sizes and shapes. However, this technique has not been applied in breast cancer treatment plan.

Based on the anatomical locations of the breast and RLN PTVs, we designed an IMRT with FJT (FJT-IMRT) where the PTV was separated into two regions, the periclavicluar and breast/IMN. The goal of the study is to compare three modulated planning techniques in the left-sided breast cancer treatment, i.e., tangential-IMRT, VMAT and FJT-IMRT, especially for thyroid and shoulder dose sparing.

## RESULTS

**Figure 1:**
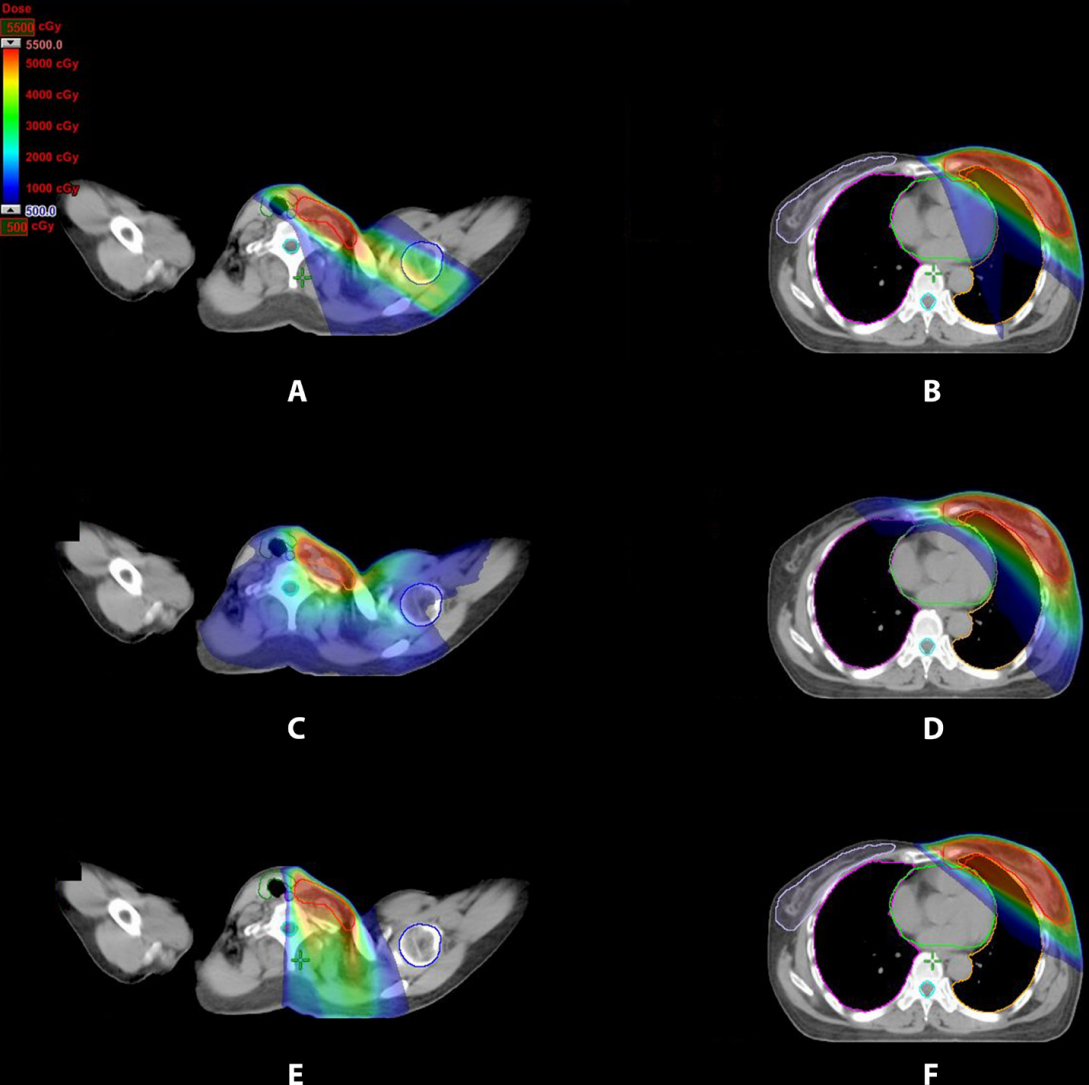
Dose distribution for three treatment plans for periclavicular node, left breast and IMN region for one representative patient (**A**, **B**) tangential-IMRT (**C**, **D**) VMAT (**E**, **F**) FJT-IMRT.

Treatment plans generated using the three techniques tangential-IMRT, VMAT and FJT-IMRT for 16 left-sided breast cancer patients previously treated with radiotherapy after lumpectomy. The dose distributions in the periclavicular and the breast/IMN regions for one representative patient were illustrated in Figure 1. VMAT plan showed low dose irradiation for all OARs, including the contralateral breast and the contralateral lung. Compared with tangential-IMRT and VMAT, FJT-IMRT had avoided irradiating part of the thyroid gland and spared the entire humeral head.

The dosimetric parameters for all plans averaged over 16 patients in this study were shown in Table 1. All three plans achieved at least 95% PTV coverage. Dose conformity was significantly improved using VMAT. In terms of the percent heart volume receiving 30 Gy (V_30 Gy_) and heart mean dose (D_mean_), tangential-IMRT and FJT-IMRT had comparable average heart V_30 Gy_ (1.5% and 1.6%) (*p >* 0.05), and VMAT further lowered the heart V30 Gy by 0.8% compared to tangential-IMRT plan (*p <* 0.01). For the average heart D_mean_, FJT-IMRT plans produced the lowest D_mean_ and it was lower than VMAT by 0.6 Gy (*p <* 0.01). For ipsilateral lung, VMAT plans increased the percent lung volume receiving 5 Gy (V_5 Gy_), although the V_30 Gy_ of VMAT was decreased. However, the contralateral breast and the contralateral lung dose from VMAT, although the value was relatively low, significantly higher than that from tangential-IMRT and FJT-IMRT.

**Table 1:** Comparison of average dosimetric parameters and number of MU of 16 patients for different planning techniques

Structure and parameter	Tangential-IMRT	VMAT	FJT-IMRT
PTV			
V_95%_ (%)	99.5 ± 0.3 (98.9–9 9.7)	99.2 ± 0.2 (99.0–9 9.5)	99.4 ± 0.3 (98.92–9 9.8)
D_2%_ (Gy)	54.4 ± 0.4 (53.9–5 5.4)	55.5 ± 0.4 (55.3–5 6.6)^a^	54.9 ± 0.6 (53.8–5 6.0)^ab^
CI	0.56 ± 0.07 (0.44–0 .69)	0.73 ± 0.03 (0.68–0 .79)^a^	0.56 ± 0.06 (0.47–0 .67)^b^
Heart			
V_5 Gy_ (%)	30.3 ± 7.7 (16.5–4 8.6)	23.4 ± 7.6 (12.8–3 6.2)^a^	15.3 ± 5.1 (6.8–2 2.6)^ab^
V_20 Gy_ (%)	5.1 ± 3.5 (1.3–1 3.4)	2.4 ± 1.8 (0.9–1 0.5)^a^	4.3 ± 3.1 (0.4–7 .1)^b^
V_30 Gy_ (%)	1.5 ± 1.1 (0.2–4 .4)	0.8 ± 0.9 (0.0–3 .5)^a^	1.6 ± 1.2 (0.2–4 .9)^b^
D_mean_ (Gy)	5.6 ± 1.3 (3.6–8 .2)	4.6 ± 1.0 (3.2–6 .6)^a^	4.0 ± 1.2 (2.3–5 .9)^ab^
Ipsilateral lung			
V_5 Gy_ (%)	72.2 ± 4.5 (63.3–8 2.3)	73.8 ± 4.6 (68.9–8 5.6)	55.1 ± 5.0 (46.0–6 3.6)^ab^
V_20 Gy_ (%)	30.4 ± 2.5 (23.1–3 2.8)	30.3 ± 1.9 (25.6–3 2.1)	30.5 ± 3.0 (23.6–3 4.8)
V_30 Gy_ (%)	23.8 ± 2.7 (17.1–2 7.1)	20.9 ± 2.0 (17.1–2 3.7)^a^	24.3 ± 3.0 (18.0–2 9.0)^b^
D_mean_ (Gy)	17.0 ± 1.5 (13.9–1 9.5)	16.7 ± 0.7 (15.3–1 7.9)	16.1 ± 1.7 (13.0–1 9.3)^a^
Contralateral lung			
V_5 Gy_ (%)	0.1 ± 0.1 (0.0–0 .3)	26.6 ± 11.3 (11.4–5 5.3)^a^	0.1 ± 0.1 (0.0–0 .4)^b^
D_mean_ (Gy)	0.5 ± 0.1 (0.4–0 .6)	3.6 ± 0.8 (2.6–5 .8)^a^	0.5 ± 0.1 (0.3–0 .6)^b^
Contralateral breast			
V_5 Gy_ (%)	0.8 ± 1.3 (0.0–4 .6)	10.3 ± 3.2 (4.8–1 5.3)^a^	0.8 ± 1.2 (0.0–4 .0)^b^
D_mean_ (Gy)	0.6 ± 0.2 (0.3–1 .1)	2.8 ± 0.2 (2.6–3 .5)^a^	0.5 ± 0.2 (0.3–0 .9)^ab^
Thyroid			
V_5 Gy_ (%)	91.9 ± 12.9 (57.3–1 00.0)	88.7 ± 11.8 (61.7–1 00.0)	48.1 ± 16.0 (27.9–1 00.0)^ab^
D_mean_ (Gy)	29.6 ± 5.3 (21.8–3 6.7)	19.1 ± 4.0 (13.2–2 8.9)^a^	16.1 ± 4.4 (7.3–2 5.9)^ab^
Humeral head			
V_5Gy_ (%)	86.3 ± 13.3 (63.4–1 00.0)	82.4 ± 15.9 (46.2–9 9.5)	22.2 ± 14.3 (2.4–4 1.4)^ab^
D_mean_ (Gy)	23.8 ± 8.2 (12.1–3 4.7)	9.0 ± 1.8 (5.7–1 1.9)^a^	4.3 ± 1.5 (2.2–6 .5)^ab^
Cervical esophagus			
V_5 Gy_ (%)	83.1 ± 14.0 (48.8–9 8.9)	98.8 ± 2.6 (92.2–1 00.0)^a^	63.6 ± 20.2 (12.1–9 2.2)^ab^
D_mean_ (Gy)	16.5 ± 5.2 (6.8–2 5.1)	17.1 ± 3.7 (10.7–2 4.7)	12.1 ± 5.3 (3.6–2 2.8)^ab^
Spinal cord			
Max (Gy)	3.5 ± 1.5 (2.0–6 .4)	24.0 ± 5.3 (13.5–3 5.3)^a^	17.1 ± 7.6 (4.1–3 0.0)^ab^
Non-target tissue			
V_5 Gy_ (%)	24.1 ± 1.5 (22.2–2 7.3)	37.4 ± 2.7 (32.9–4 1.9)^a^	19.2 ± 1.5 (15.6–2 1.7)^ab^
D_mean_ (Gy)	6.3 ± 0.5 (5.6–7 .4)	7.9 ± 0.5 (7.3–8 .8)^a^	5.8 ± 0.4 (5.2–6 .8)^ab^
#Monitor unit	952.0 ± 223.9(673.0–1 509.0)	428.9 ± 31.3 (380.0–4 99.0)^a^	1348.1 ± 393.8(901.0–2 195.0)^ab^

Abbreviations: D_2%_ = near-maximum dose, CI = conformity index, V_xGy_ = the percent volume of the structure receiving xGy, D_mean_ = mean dose. Data presented as mean ± standard deviation, range between brackets.

^a^Significantly different from tangential-IMRT.

^b^Significantly different from VMAT.

**Figure 2:**
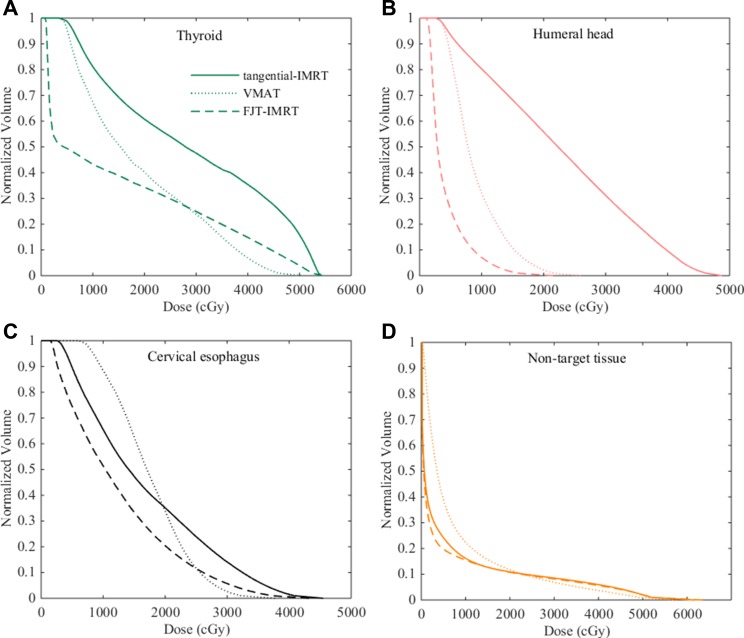
Dose volume histograms with different planning techniques for one representative patient (**A**) Thyroid (**B**) Humeral head (**C**) Cervical esophagus (**D**) Non-target tissue.

The mean dose–volume histograms (DVHs) of thyroid, humeral head and cervical esophageal were displayed in Figure 2. Compared with tangential-IMRT and VMAT plans, FJT-IMRT reduced the mean dose of thyroid, humeral head and cervical esophageal by 47.6% (*p <* 0.01) and 45.7% (*p <* 0.01), 74.3%(*p* = < 0.01) and 73% (*p* = < 0.01), and 26.7% (*p* = < 0.01) and 29.2% (*p* = < 0.01).

The mean number of monitor unit for FJT-IMRT plans was 1348.1±393.8 compared with 952 ± 223.9 for tangential-IMRT (*p <* 0.01) and 428.9 ± 31.3 for VMAT (*p <* 0.01). However, a slight decrease in the dose to non-target tissues was observed in FJT-IMRT plans compared to those using other two techniques (Table 1 and Figure 2D).

## DISCUSSION

In this study we compared three techniques: tangential-IMRT, VMAT, and FJT-IMRT for locoregional radiotherapy of left-sided breast cancer. We demonstrated FJT-IMRT has sufficient PTV coverage and superior OARs sparing (especially for thyroid, shoulder, and cervical esophagus) to tangential IMRT and VMAT. To the best of our knowledge, there is no published study to evaluate the dose of OARs in the periclavicular region.

VMAT plans achieved the lowest heart V_30 Gy_ at the cost of increased dose to the contralateral breast and the contralateral lung. Similar to other studies [7, 10], this study also found that VMAT plans could reduce the high dose volume of the heart as well as the dose to the contralateral breast and the contralateral lung. However, the dose to the contralateral breast may expose patients under the age of forty years old to increased risk of developing a secondary malignancy [11, 12], therefore, weather using VMAT technique especially for those young patients should be considered with the risk benefit ratio in mind. On tangential-IMRT and FJT-IMRT plans, the beams were orientated in such a way to avoid going directly toward the contralateral breast and the contralateral lung, and as a compromise slightly more heart volume was irradiated. Consequently tangential-IMRT could be optimal in these situations as it could reduce the radiation exposure to the OARs and in the meantime maintain adequate PTV coverage [9, 13].

It was reported by Dogan et al. [9] that the radiation doses to the thyroid, esophagus and humeral head were increased with tangential-IMRT for locoregional radiotherapy. In another study published by Jagsi et al. [13] the treatment target was divided into periclavicular and breast/ IMN regions, and the integrated treatment plan was optimized in complicated steps. Thyroid and shoulder were not contoured as OARs and doses to these structures were not evaluated. We used a similar radiation field design in FJT-IMRT plans as in their studies [9, 13]. Compared with tangential-IMRT, FJT-IMRT plans generated similar heart V_30 Gy_ but with significantly lower D_mean_. Furthermore, FJT-IMRT achieved more dose sparing for the thyroid, shoulder and cervical esophagus.

The thyroid is a radiosensitive organ. Several authors reported a 16 to 48% risk of developing hypothyroidism [15–19]. The potential impact factors for hypothyroidism after radiation or chemoradiotherapy in these studies included the thyroid volume, mean thyroid dose, percent volume of thyroid receiving high dose irradiation (30 Gy to 50 Gy) and gender. Marianne et al. [19] developed a normal tissue complication probability (NTCP) model for radiation-induced biochemical hypothyroidism after primary radiotherapy for head and neck carcinoma. With the latent time integrated in their NTCP model, they found thyroid D_mean_ and volume were the independent risk factors for hypothyroidism. Therefore, limiting the thyroid dose in treatment plan may potentially reduce hypothyroidism in breast RLN irradiation. In conventional breast locoregional irradiation, thyroid is often considered as an organ at low risk. Two authors reported the thyroid received increased dose from 3DCRT with single anterior field [22, 23] for breast locoregional irradiation. Our study demonstrated that tangential-IMRT plans actually increased thyroid Dmean and FJT-IMRT offered the most protection for thyroid. Both VMAT and FJT-IMRT produced similar thyroid Dmean, which compared to tangential-IMRT plans was significantly reduced by as much as 46% for VMAT and 35% for FJT-IMRT, respectively.

Postoperative adjuvant irradiation with or without axillary irradiation could increase the risks of shoulder restriction, upper limb weakness, lymphedema and pain which reduced the quality of life [24, 25]. Therefore avoiding irradiation of the shoulder and/or axillary can greatly decrease the arm and shoulder symptoms. Our study has shown that the humeral head Dmean was the highest on tangential-IMRT plans and the lowest with FJT-IMRT, therefore, FJT-IMRT was the optimal choice among all three techniques to minimize the dose to the patient's shoulder area. Furthermore high dose irradiation of the cervical esophagus could cause discomfort and pain when patients swallow. Since our data suggested that FJT-IMRT had the lowest Dmean for cervical esophagus, it is clear that FJT-IMRT is also the optimal treatment technique for locoregional breast cancer especially for certain subgroups of the patients, such as young left breast cancer patients and/or patients with previous thyroid disease.

Although FJT-IMRT produced high quality treatment plans, one drawback of this technique was the decreased delivery efficiency. The FJT-IMRT plans in general tend to have large number of MU. For beams at 600 MU/min dose rate, FJT-IMRT could require additional 1 to 2min of treatment time. However this increased beam-on time is generally insignificant and will not cause any practical issues in patient comfort and throughput.

We did not use any breath hold technique for the left-sided breast irradiation in our study. It was demonstrated in many publications that inspiration could reduce the cardiac volume exposed to radiation as the heart typically moves away from the chest wall [28, 29]. Korreman and colleagues estimated the median cardiac mortality NTCP for breath hold treatment to be 0.1% and concluded that breath hold and respiratory gating techniques could reduce mortality by 4.7% compared with free breathing treatment in the left breast cancer patients [30]. However, in our study the main focus was on the dose sparing of the thyroid and shoulder. Since the effect of respiratory motions on the doses to the thyroid and shoulder are minimal, breath hold was not used in the dosimetric comparison of FJT-IMRT, VMAT and tangential-IMRT techniques.

## MATERIALS AND METHODS

### Patient data

Sixteen patients with left-sided invasive breast cancer who received radiotherapy after BCS in our institution were randomly selected for this study. These patients represented a range of breast sizes and body habitus. The patients were placed in supine position on a breast tilt board (Med-Tech 350) to make sternum parallel to the table with both arms fully abducted (90°or greater) and externally rotated. A planning CT with slice thickness of 5mm was acquired from mid-neck to diaphragm with no contrast for each patient on a large bore CT-simulator (Brilliance CT, Philips, Cleveland, OH, USA). This investigation was approved by the Institutional Review Board.

### Definition of PTVs and OARs

The clinical target volumes (CTV) consisted of the ipsilateral breast, IMN and periclavicular nodes for each patient. The breast and RLN CTV were delineated according to consensus definition given by the Radiation Therapy Oncology Group breast cancer atlas for radiotherapy. The planning target volumes (PTV) were generated by adding a margin of 5 to 10mm to the CTV except for superficial region where PTV was limited to 5mm inside the skin surface.

The OARs included the bilateral lungs, heart, contralateral breast, spinal cord, cervical esophagus, thyroid and shoulder. To assess the dose to the shoulder, the left humeral head was contoured. The heart was contoured from the level of pulmonary trunk superiorly to the apex, including the pericardium but not the major vessels. And the cervical esophagus was contoured from cricoid cartilage to thoracic inlet. In addition, the non-target tissue was defined as the patient's body excluding PTV.

### Treatment planning

For each patient, three treatment plans using tangential-IMRT, VMAT and FJT-IMRT techniques were created on the Eclipse treatment planning system (version 11, Varian Medical Systems, Palo Alto, CA). The same isocenter and 6MV photon beams on a Varian TrueBeam linear accelerator were used for all the plans.

**Figure 3:**
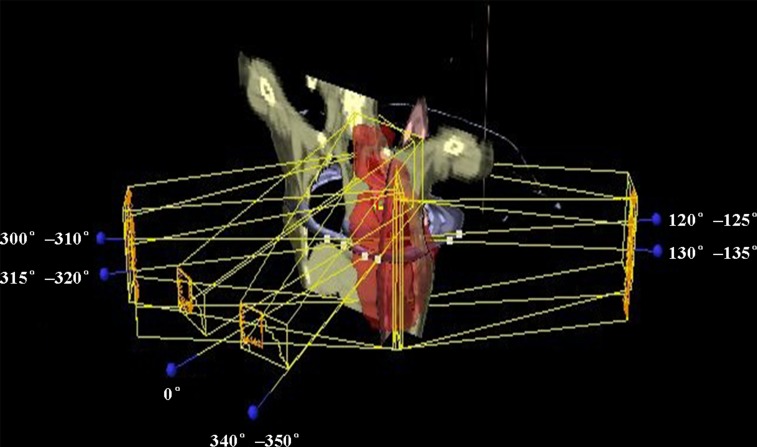
Beam arrangement for FJT-IMRT beam angles with 0° and 340°–350° for periclavicular region, beam angles with 300°–310° and 315°–320°for medial tangential direction and beam angles with 120°–125° and 130°–135°for lateral tangential direction.

For the tangential-IMRT plan, the beam placement was limited to the left breast to avoid the dose to the contralateral breast and the contralateral lung. Five gantry angles were used, i.e., (300°–310°), (315°–320°), (340°–350°), (120°–125°) and (130°–135°). The collimator jaw positions were automatically set to cover the entire PTV. For the FJT-IMRT planning, the PTV was divided into two regions at caudal edge of clavicular head for dose optimization. The beam arrangement was depicted in Figure 3. Four beam angles similar to those in tangential-IMRT were selected for the breast/IMN region PTV IMRT, i.e., (300°–310°), (315°–320°), (120°–125°) and (130°–135°), and the superior collimator jaw position was fixed and set to the target split line. For the periclavicular region PTV, we used two beam angled at (340°–350°) and 0° and the inferior collimator jaw position was fixed to target split line. The third plan used the VMAT technique with two 6MV partial arcs, the starting gantry angle was set to between 290°and 300°, and the stop angle between 135°and 180°. The collimator was rotated to 30°and 330°for the two arcs [7].

### Plan optimization and dose constraints

In the optimization process, the dose constraints which were derived from the clinical experience in our institution and from the published studies, are listed as follows. At least 95% of the PTV receives 50 Gy; No more than 2% of heart can receive more than 30 Gy (V_30 Gy_ ≤ 2%) and mean heart dose less than 5 Gy (D_mean_ ≤ 5 Gy); For the ipsilateral lung, V_20 Gy_ ≤ 30% and D_mean_ ≤ 20 Gy; The maximum spinal cord dose should be less than 45 Gy; For the cervical esophagus D_mean_ ≤ 30 Gy and V_5 Gy_ ≤ 80%; For thyroid D_mean_ ≤ 25 Gy and V_5 Gy_ ≤ 80%; For humeral head D_mean_ ≤ 25 Gy and V_5 Gy_ ≤ 80%; For the contralateral breast D_mean_ ≤ 1 Gy and V_5Gy_ ≤ 30%; And for the contralateral lung D_mean_ ≤ 1 Gy and V_5 Gy_ ≤ 30%. The first priority in optimization was to meet the PTV objective. The second priority was to satisfy the dose constrains for the heart, ipsilateral lung and contralateral breast. During the plan optimization, dose constrains in some cases were relaxed in order to achieve homogeneous dose distribution in PTV. The dose was computed with the anisotropic analytical algorithm for all cases and the dose calculation grid was set to 2.5 mm.

### Dosimetric evaluation and statistical analysis

DVHs were generated for PTV and all OARs. For the PTV, the percent of the volume receiving more than 95% of prescribed dose (V_95%_), the maximum dose D_2%_ which represented the highest dose received by 2% of the volume were reported. The conformity index (CI) was defined as CI = TV_RI_/TV * TV_RI_/V_RI_, where TV_RI_ was the target volume covered by the reference dose (95% of the prescribed dose), TV was the target volume, and V_RI_ was the volume of the reference dose. For OARs, such as the heart, bilateral lungs and humeral head, thyroid, cervical esophagus, contralateral breast, and non-target tissue (body-PTV), V_xGy_ and D_mean_ were calculated and compared. The monitor units from each plan were also reported. Statistical analysis was performed using the paired two-tailed Student's t test and a significance level of 0.05. The statistical significance of the differences was tested for the tangential-IMRT against FJT-IMRT and VMAT plans.

## CONCLUSIONS

Three treatment techniques for the left breast cancer locoregional radiotherapy in this dosimetric study were two partial arc VMAT, tangential-IMRT and FJT-IMRT. All three techniques provided similar adequate PTV coverage. The reduced heart V30 Gy indicated that VMAT could improve dose sparing to the heart, however, the dose to the contralateral breast and the contralateral lung was slightly increased. On the other hand, the tangential-IMRT and FJT-IMRT plans had slightly higher heart V30 Gy but minimized the irradiation of the contralateral breast and the contralateral lung. Furthermore the FJT-IMRT technique decreased doses to the thyroid, humeral head and cervical esophagus. For certain patient subgroups, especial for young left-sided breast cancer patients under forty years old and for those patients with previous thyroid diseases, FJT-IMRT could be the optimal treatment technique.

## Abbreviations

BCS: breast conservative surgery
RLN: regional lymph node
IMN: internal mammary node
3DCRT: three-dimensional conformal radiotherapy
IMRT: intensity modulated radiotherapy
VMAT: Volumetric modulated arc therapy
Tangential-IMRT: tangential multibeam IMRT
OAR: organ at risk
FJT: fixed collimator jaw technique
FJT-IMRT: IMRT with FJT
V_xgy_: percent volume receiving xGy
D_mean_: mean dose
NTCP: normal tissue complication probability
CTV: clinical target volumes
PTV: planning target volume
V_95%_: percent volume receiving ≥ 95% of prescribed dose
D_2%_: the highest dose received by 2% of the volume
CI: conformity index
TV_RI_: target volume covered by the reference dose (95% of the prescribed dose)
TV: target volume
V_RI_: volume of the reference dose
